# The role of a behavioural medicine intervention in physiotherapy for the effects of rehabilitation outcomes in exercise-based cardiac rehabilitation (ECRA) – the study protocol of a randomised, controlled trial

**DOI:** 10.1186/s12872-017-0557-7

**Published:** 2017-05-25

**Authors:** Sabina Borg, Birgitta Öberg, Lennart Nilsson, Anne Söderlund, Maria Bäck

**Affiliations:** 1 Department of Medical and Health Sciences, Division of Physiotherapy, Linköping University, SE-581 83 Linköping, Sweden; 2 Department of Cardiology and Department of Medical and Health Sciences, Linköping University, Linköping, Sweden; 3 Department of Medical and Health Sciences, Division of Cardiovascular Medicine, Linköping University, Linköping, Sweden; 4 Department of Physiotherapy, School of Health, Care and Social Welfare, Mälardalen University, Västerås, Sweden

**Keywords:** Coronary artery disease, Feedback, Goal-setting, Physical fitness, Physiotherapy, Self-efficacy, Self-monitoring

## Abstract

**Background:**

To help patients with coronary artery disease (CAD) benefit from the positive health effects attained by exercise-based cardiac rehabilitation (CR), adherence to these programmes according to international guidelines is important. Strategies to increase adherence to exercise-based CR are mainly an unexplored area. The objective of this study is to investigate the effects of a behavioural medicine intervention in physiotherapy, containing goal-setting, self-monitoring and feedback, with the aim of improving rehabilitation outcomes for exercise-based CR, compared with usual care.

**Methods:**

This is a randomised, controlled trial. A total of 160 patients with CAD will be included consecutively at the Coronary Care Unit at a university hospital in Sweden. Patients are randomised 1:1 using sealed envelopes to usual care or a behavioural medicine intervention in physiotherapy, in addition to usual care for 4 months. Outcome assessment at baseline, 4 and 12 months includes submaximal aerobic capacity (primary outcome), exercise adherence, muscle endurance, level of physical activity, biomarkers, anxiety and depression, health-related quality of life, patient enablement and self-efficacy (secondary outcomes).

**Discussion:**

This is the first study to evaluate the role of an integrated behavioural medicine intervention in exercise-based CR in the effects of rehabilitation outcomes. The results of this study will provide valuable information about the effect of these interventions in exercise-based CR and it has the potential to inform and assist in further treatment in secondary prevention for patients with CAD.

**Trial registration:**

The study include all items from the World Health Organization Trial Registration Data Set. Trial registration number: NCT02895451, 2016-08-16, retrospectively registered.

## Background

Coronary artery disease (CAD) causes a large amount of disability and death in western countries, placing a substantial burden on the health-care systems and the economy [1]. One primary driver of the large costs to the health-care system is rehospitalisation due to repeated CAD events. During the last few decades, mortality rates from CAD have decreased and this can be attributed to improved medical treatment and the control of traditional cardiovascular risk factors [2]. As a result, candidates in need of secondary prevention are growing in number.

Secondary prevention in CAD, administered through cardiac rehabilitation (CR), is the main contributor to the mortality reduction [3]. CR has been defined as the “coordinated sum of interventions required to ensure the best physical, psychological and social conditions so that patients with chronic or post-acute cardiovascular disease may, by their own efforts, preserve or resume optimal functioning in society and, through improved health behaviors, slow or reverse the progression of disease” [4, 5]. International guidelines consistently identify exercise as a cornerstone of CR [4, 5]. Physiotherapists play an important role in exercise-based CR when it comes to prescribing individually tailored exercise programmes. Exercise is defined as *“a subset of physical activity that is planned, structured, repetitive, and purposeful in the sense that the improvement or maintenance of physical fitness is the objective”* [6]. International guidelines have established recommendations for aerobic and resistance exercise in the secondary prevention of CAD [4].

Meta-analyses clearly confirm the benefits of exercise-based CR in terms of marked reductions in cardiovascular mortality, as well as a reduced risk of hospital admission [7, 8]. Furthermore, exercise-based CR has favourable effects on cardiovascular risk markers [8] and aerobic exercise capacity [7], as well as on anxiety and depression [9]. As peak aerobic exercise capacity is a strong predictor of mortality in patients with CAD, a small gain in oxygen uptake may improve not only functional capacity but also survival prospects [10]. In order to obtain these positive health effects, adherence to exercise-based CR programmes in accordance with international guidelines for patients with CAD is important [4, 5].

Despite the well-established positive effects of exercise-based CR, adherence in patients with CAD remains low in many countries [11, 12]. International studies have found that fewer than a third adhere to the prescribed exercise programme [13] and another study found that fewer than 50% maintain their exercise habits 6 months after completing an exercise-based CR programme [12]. In Sweden in 2016, the average participation rate for exercise-based CR in patients with CAD was 52% [14].

Strategies to increase adherence to exercise-based CR are still a partially unexplored area. A recent Cochrane Review concluded that there is weak evidence for the effect of interventions aiming to increase adherence to exercise-based CR and no clinical recommendations could be made [12]. There was a limited ability to determine the consistency of findings due to varied and multifaceted interventions and meta-analysis was not possible due to heterogeneity. Data on important clinical outcomes such as cardiovascular risk factors were lacking and none of the studies found a difference in health-related quality of life. In conclusion, none of the studies was evaluated as having a low risk of bias and further high-quality research is needed [12].

In the area of behavioural medicine, it has previously been shown that self-monitoring is the most important component for a behavioural change to be successful in healthy adults when changing exercise behaviour [15]. Interventions that combined self-monitoring with at least one other component, such as goal-setting or feedback on behaviour/exercise, proved to be more effective than other interventions that were evaluated [15]. There is, however, a need to evaluate the effects of these interventions in the context of adherence to exercise-based CR.

### Objectives

The objective of this study is to investigate the effects of a behavioural medicine intervention in physiotherapy, containing goal-setting, self-monitoring and feedback, with the aim of improving rehabilitation outcomes for exercise-based CR, compared with usual care at 16 weeks (end of the intervention) and 12 months after the index event (follow-up).

#### Primary objective

The primary objective is to investigate the effect of a behavioural medicine intervention in exercise-based CR on submaximal aerobic exercise capacity, compared with usual care. (Sample size calculations are performed based on differences in submaximal aerobic exercise capacity at 16 weeks.)

#### Secondary objectives

The secondary objectives are to evaluate the effects of a behavioural medicine intervention in exercise-based CR on exercise adherence, muscle endurance, level of physical activity, physiological parameters, anxiety and depression, health-related quality of life, patient enablement and self-efficacy, compared with usual care.

## Methods

### Study design

This is an open-label, randomised, controlled trial.

### Subjects

A sample of 160 patients will be consecutively included at the Coronary Care Unit (CCU) at Linköping University Hospital, Sweden. The inclusion criteria are a primary care event due to CAD and/or percutaneous coronary intervention (PCI), age < 75 years. The exclusion criteria are serious physical or psychological disease interfering with participation in exercise-based CR and an inability to understand the Swedish language. The study process will be presented in accordance with the CONSORT guidelines of a parallel randomised trial, including enrolment, intervention allocation, follow-up and data analysis [16].

### Procedure

A pilot study comprising five patients with CAD was completed prior to the study to ensure the quality of the test procedure and to test the behavioural medicine intervention. Based on the results of the pilot study, small modifications to the behavioural medicine intervention protocol were made, including a clarification of the wording in the study protocols.

The physiotherapist at the CCU receives daily information about patients eligible for the study, asks potential patients to participate and obtains informed consent. An appointment with a physiotherapist, responsible for the testing procedure, at the exercise-based CR takes place within 2 to 3 weeks after discharge to perform baseline testing. The test procedure includes measurements, described more specifically below, and takes approximately 1.5 h. The patients are asked to refrain from strenuous exercise 24 h before the test and not to consume caffeine or nicotine for 2 h before the testing session. After finishing the baseline tests, the physiotherapist randomise patients to either routine care or the behavioural medicine intervention, in addition to usual care, using sealed envelopes. Randomisation will be stratified by submaximal aerobic work capacity with a cut-off at 100 watts, based on previous clinical data from a similar exercise-based CR setting (*n* = 50) and clinical experience from the present exercise-based CR setting. Patients randomised to usual care follow routine exercise-based CR care, as described below. Patients in the intervention group receive the behavioural medicine intervention, in addition to usual care. No co-intervention bias is identified. Adverse events will be reported according to the study protocol. No post-trial care will be provided. Due to organizational circumstances, the physiotherapists performing the tests are not blinded to group allocation.

### Intervention

#### Usual care

Usual care in exercise-based CR is based on international guidelines [4, 5]. The exercise programme is individually prescribed, based on the results of tests of physical capacity. The exercise programme consists of hospital-based aerobic exercise twice a week with a duration of 20–60 min and an intensity of 40-80% of Vo2max, depending on each patient’s physical fitness. The aerobic exercise is complemented with resistance exercise; 8-10 different upper and lower body exercises, 1-3 sets of 10-15 repetitions. In addition, patients are instructed to perform one home-based aerobic exercise session. Some patients choose to perform their entire exercise-based CR in a home-based setting. Patients register their home-based exercise sessions in an exercise diary, which is not followed up by the physiotherapist during the exercise-based CR period. No other structured interventions to increase or control exercise adherence are included in usual care.

#### Behavioural medicine intervention

In addition to exercise-based CR according to usual care, patients in the intervention group receive a behavioural medicine intervention aimed at increasing adherence to the prescribed exercise programme. The behavioural change techniques used in the current study, i.e. specific goal setting, re-evaluation of the goals, self-monitoring and feedback, are based on control theory (CT) [17]. CT provides a model of self-regulation that is useful in the analysis of human behaviour. This model describes the process of self-regulation as involving the self-imposition of behavioural standards, observation of one’s own actions and evaluation of the actions by comparing them with the standards [17]. CT is part of the Social Cognitive Theory of Self-Regulation [18], which also highlights the importance of self-efficacy in order to change a behaviour [19]. Self-monitoring and goal-setting are central behavioural change techniques supported by the Social Cognitive Theory of Self-Regulation and are important factors in the process of self-regulation. Self-monitoring provides the information needed to set realistic goals and evaluate progress towards these goals [19].

##### Specific goal-setting and re-evaluation of goals

The behavioural medicine intervention starts with an appointment with a physiotherapist for detailed planning and goal-setting for the exercise-based CR. The exercise goal is set following agreement between the patient and the physiotherapist, based on activities the patient believes are achievable. Potential facilitators and barriers in relation to achieving the exercise goals are identified and discussed and an action plan with strategies is drawn up together with the patient. Specific goal-setting according to CT involves the detailed planning of what the person wants to do, including a definition of the behaviour by specifying what to do, including exercise frequency, intensity, duration and specification of the context in which the exercise is done [20]. The patient’s motivation and self-efficacy for initiating and maintaining a physical activity-related behavioural change is self-rated using a visual analogue scale (VAS) and is discussed in relation to goal-setting. The exercise goal is re-evaluated during the intervention period. It has been found that reviewing and/or reconsidering previously set goals during the change process is important according to CT [20]. Previous studies [13, 15, 21] have pointed out that the physiotherapist can play an important role when it comes to providing feedback on potential barriers to exercise, as well as the reformulation of goals [13, 15, 21].

##### Self-monitoring and feedback

In the current study, the patients are requested to self-monitor their defined exercise goal by completing an exercise diary. This exercise diary includes a specification of the performed exercise dose (frequency, intensity and duration) and it is followed up by a physiotherapist every 3 weeks by a phone call or a personal meeting. The aim of the feedback is to monitor the patient’s progress at each contact, provide positive feedback on achieved goals, give supporting feedback on potential barriers to exercise and discuss strategies to increase adherence. In addition, visual feedback on physical activity level is reported by accelerometry at 9 weeks (after having performed half the exercise-based CR programme). Accelerometry has been shown to be reliable and valid for measuring physical activity and sedentary time and providing detailed information on intensity, frequency and duration, without reporting errors associated with self-reports [22]. Researchers have determined accelerometer cut-points in calibration studies, which make it possible to classify the measured physical activity into the different intensity levels of light, moderate and high [23]. Even though the accelerometer cut-points are not calibrated for each individual, the graphically presented feedback has been shown in previous studies to increase adherence, by helping the patient to visualise the performed physical activity in comparison with the achievable goals [24]. This makes it easier for the person to see the need for improvement.

At the end of the intervention (16 weeks), the patient is offered a follow-up meeting with the physiotherapist. This meeting will offer the patient an opportunity to express his/her perceptions of the intervention and how the goal-setting was achieved. Moreover, a long-term exercise goal is set and potential future barriers, facilitators and strategies can be brought up and discussed in order to facilitate future adherence to exercise.

To summarise, the behavioural medicine intervention contains one initial meeting (baseline), three structured telephone/personal follow-ups (3, 6 and 12 weeks), one personal meeting for visual feedback with an accelerometer after 2 months (9 weeks) and one meeting at the end of the intervention (16 weeks).

The assigned study intervention will be modified or discontinued if the patient experience worse health status interfering with participating in exercise-based CR or withdrawal of participant consent. Outcome data will be collected for patients who discontinue or deviate from intervention, in accordance with intention to treat design.

### Outcomes

Clinical and demographic patient characteristics are taken from medical records and patient interviews. Changes in the following variables will be measured from baseline to the first (16 weeks at end of intervention) and second (12 months after index event) follow-up visits.

#### Primary outcome

##### Submaximal aerobic exercise capacity

The primary outcome is changes in Watts (W) on the submaximal exercise test on a bicycle ergometer from baseline to the first follow-up visit (16 weeks). Blood pressure and heart rate are registered before the start of the test. The test is performed on a bicycle ergometer according to the WHO protocol [25], with an increased workload of 25 W every 4.5 min [26, 27]. The initial starting load, 25 W or 50 W, is decided, based on the patient’s exertion history. A pedalling rate of 50 rates per minute is used. After 2 and 4 min of each workload; heart rate, rating of perceived exertion according to Borg’s rating of perceived exertion scale (RPE) [28] and subjective symptoms, including chest pain and dyspnea according to Borg’s Category Ratio Scale, CR-10, scale are rated. After 3 min, the systolic blood pressure is registered. The exercise test is discontinued at Borg RPE 17 and/or dyspnea 7 on Borg’s CR-10 scale. The submaximal exercise test on a bicycle ergometer is a well-established, safe procedure that is frequently used in exercise-based CR in Sweden. The reliability of this test is currently being tested on a Swedish population with CAD.

#### Secondary outcomes

##### Muscle endurance

Muscle endurance is evaluated by two clinical endurance tests that are included in usual exercise-based CR in Sweden. They have been found to be reliable for patients with chronic heart failure [29]_._

Unilateral isotonic shoulder flexion: the patient sits comfortably on a chair, with his/her back touching the wall, holding a dumbbell (2 kg for women and 3 kg for men) in an optional hand. The patient is asked to lift his/her shoulder from 0° to 90° flexion as many times as possible, using a pace of 20 contractions per minute kept by a metronome. The number of elevations is registered.

Unilateral isotonic heel lift: the patient performs a maximum heel lift on a 10° tilted wedge, one lift every other second, with the pace kept by a metronome. The patient chooses which leg to perform the test, the contralateral foot is held slightly above the floor and, for balance, the wall is touched with the fingertips. The number of maximum heel lifts is counted.

##### Level of physical activity

Physical activity is measured over seven consecutive days with the ActiGraph GT3X-BT triaccelerometer (ActiGraph, Pensacola, FL) with normal filter settings [30]. The device firmware transforms these impulses and summarises them over discrete epochs into counts per time interval [31]. The GT3X-BT is 3 cm × 3 cm × 2 cm and weighs several grams. Measurement data conversion is performed with ActiLife 6.8.0 (ActiGraph, Penascola, FL). ActiGraph accelerometers are widely used in research because they have shown high validity and reliability [32–34].

Self-reported physical activity is measured with a diary according to Bouchard for three consecutive days. The level of physical activity is rated from 1 (sleep) to 9 (very high intensity), based on energy consumption [35].

##### Anxiety and depression

Anxiety and depression are measured using the Hospital Anxiety and Depression Scale (HADS) [36]. This scale is a 14-item self-report comprising seven anxiety items and seven depression items, from which separate anxiety and depression scores are calculated. The items are scored on a four-point Likert scale, ranging from 0 to 3 points. The HADS is considered to be reliable and valid for patients with CAD [37, 38].

##### Health-related quality of life

Health-related quality of life (HRQoL) is evaluated by the Short-Form-36 (SF-36) [39] and the EQ5D [40]. The SF-36 is a generic, psychometrically sound instrument, comprising 36 items across eight dimensions. The responses to each question within a dimension are combined to generate a score from 0 to 100, with higher scores indicating better health. The SF-36 has been found to be reliable and valid for patients with CAD [41]. The EQ5D is a standardised HRQoL instrument consisting of a descriptive part with five questions/dimensions and a visual analogue scale on which the patient rates state of health on a scale of 0-100, where 0 is the worst state and 100 the best possible state of health. The EQ5D is considered to be reliable and valid for patients with CAD [42].

##### Patient enablement

Patient enablement is evaluated through the Swedish validated version of the Patient Enablement Instrument (PEI) [43]. The PEI consists of six questions designed to reflect patient enablement, focusing on understanding and coping with health issues and illness. The answers are graded on a three-point scale ranging from 0 to 2 points. The PEI is considered to be reliable and valid for patients in a primary care setting [43].

##### Self-efficacy

Self-efficacy is evaluated through the Swedish validated version of the Self Efficacy Scale for Exercise (SEE) (Cavrak et al., unpublished data). The Swedish version of the SEE includes nine situations that could affect participation in exercise. The patient rates, on a scale from 1 to 10, his/her confidence in performing exercise. The SEE has been found to be reliable and valid for older adults [44].

##### Biomarkers

Non-fasting venous blood samples will be collected. Complete blood count, HbA1c, creatinine and lipids are analyzed on fresh samples. In addition, plasma is separated by centrifugation and collected in -70C freezer for later analysis of markers of inflammation, platelet function and apoptosis.

##### Exercise adherence

Exercise adherence is defined as “*the extent to which a patient acts in accordance with the agreed (original stated as advised) interval, exercise dose, and exercise dosing regimen”* [11]. As the intervention in this study has a patient-centered approach, it is important that the exercise dose is agreed between the patient and the physiotherapist. Exercise adherence will be evaluated using the exercise diary and accelerometer measurements.

**Fig. 1:**
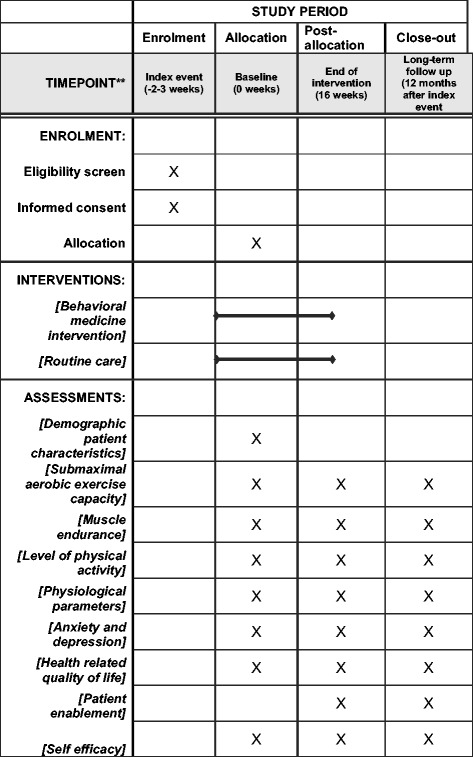
Overview of enrolment, interventions, and assessments

See Fig. 1 for an overview of enrolment, interventions, and assessments.

### Statistical methods

Data analysis will be performed using SPSS for Windows (IBM Corp. Armonk, NY). Descriptive statistics will be used for demographic data. The normally distributed continuous variables (ratio and interval data) will be presented as the mean ± 1SD. Skewed ratio and interval data, as well as ordinal data, will be presented as the median and 25th-75th percentiles. For nominal data (categorical variables), absolute and relative numbers (%) will be described.

Differences between groups will be tested with Student’s t-test for normally distributed ratios and interval data or the Mann-Whitney U test for skewed data. For comparisons of paired observations within each study group, a paired t-test (normally distributed) or Wilcoxon’s rank sum test (skewed data) will be used. For nominal data, differences within and between the groups will be calculated with a chi-square test. We will perform an-intention-to-treat and a per-protocol analysis. If adequate, missing data will be handled with multiple imputation. In addition, an analysis of predictors of rehabilitation outcomes will be calculated. A *p*-value of <0.05 will be considered statistically significant.

### Sample size calculation

Sample size calculations are based on previous clinical data from a similar exercise-based CR setting (*n* = 50) for differences in aerobic exercise capacity measured by a submaximal exercise test (Watts) before and after completed exercise-based CR. With a power of 80% and a two-sided significance level of *p* < 0.0, a least mean difference at 10 W (SD 20 W) and a calculated loss of follow-up of 20%, the estimated total sample size is 160 patients.

### Study status

The study started to recruit participants in January 2016. By March 2017, 71 patients had been included. Study inclusion is expected to be completed by 2018 and the completion of the second follow-up period is anticipated in 2019.

## Discussion

This is the first study to investigate the effects of a behavioural medicine intervention in physiotherapy, containing goal-setting, self-monitoring and feedback, on improvements in rehabilitation outcomes for exercise-based CR, compared with usual care. Despite the positive effects of exercise-based CR, adherence is sub-optimal in many countries [12, 14]. Given the stepwise benefits of greater programme participation, patients should not only be referred to and attend programmes, they should also participate as regularly as possible, according to international guidelines [4], to achieve the best possible health benefits [45].

One strength of the present study is that the behavioural medicine intervention that is used is grounded in a theoretical framework that is evidence based in other patient groups [46, 47]. The intervention is expected to be possible to implement in the existing exercise-based CR models. Another strength of the study is the long-term follow-up, making it possible to analyse the sustained effects of the behavioural change supporting intervention over time.

The limitations of the study include the single-centre design, which may affect the generalisability of the results. Moreover, because of organisational factors, it is not possible to blind the physiotherapists performing the tests to group allocation. However, the test protocols are standardised and the test procedure is validated between the physiotherapists that are involved. Another challenge in the study might be that the patients in the usual care group and the intervention group participate in the same exercise-based CR sessions at hospital. The physiotherapists that are involved are, however, strictly informed not to give any information about the behavioural medicine intervention during these exercise sessions.

To conclude, this paper presents the protocol and data analysis plan for an open-label, single-centre, randomised clinical study evaluating the role of an integrated behavioural medicine intervention in exercise-based CR in the effects of rehabilitation outcomes. When completed, the results of this study will provide valuable information about the effect of these interventions in exercise-based CR and it has the potential to inform and assist in further treatment in secondary prevention for patients with CAD.

## Abbreviations

CAD: Coronary artery disease
CCU: Coronay Care Unit
CR: Cardiac rehabilitation
CT: Control theory
HADS: Hospital Anxiety and Depression Scale
HRQoL: Health-related quality of life
PEI: Patient Enablement Instrument
SEE: Self Efficacy Scale for Exercise
VAS: Visual analogue scale
